# Brain Perivascular Space Morphometry Reflects Shared Familial Microvascular Architecture and Neuropsychiatric Influences

**DOI:** 10.1161/STROKEAHA.126.055378

**Published:** 2026-07-17

**Authors:** Alexandra Morozova, Maria del C. Valdés Hernández, Roberto Duarte Coello, Heather C. Whalley, Andrew McIntosh, Anca-Larisa Sandu Giuraniuc, Gordon D. Waiter, Christopher J. McNeil, J. Douglas Steele, Jennifer A. Macfarlane, Alison D. Murray, Joanna M. Wardlaw

**Affiliations:** Centre for Clinical Brain Sciences, Institute for Neuroscience and Cardiovascular Research, College of Medicine and Veterinary Medicine, The University of Edinburgh, United Kingdom (A. Morozova, M.C.V.H., R.D.C., H.C.W., A.M., J.M.W.).; Department of Anatomy, Third Faculty of Medicine, Charles University, Prague, Czechia (A. Morozova).; UK Dementia Research Institute at The University of Edinburgh, Edinburgh, United Kingdom (M.C.V.H., R.D.C., J.M.W.).; Scottish Imaging Network: A Platform for Scientific Excellence (SINAPSE, www.sinapse.ac.uk) (M.C.V.H., R.D.C., H.C.W., A.-L.S.G., G.D.W., C.J.M.N., J.D.S., J.A.M., A.D.M., J.M.W.).; School of Medicine, Medical Sciences and Nutrition, University of Aberdeen, United Kingdom (A.-L.S.G., G.D.W., C.J.M.N., A.D.M.).; School of Medicine, University of Dundee, Ninewells Hospital, Dundee, United Kingdom (J.D.S.).; NHS Tayside 1251 , Dundee, United Kingdom (J.A.M.).; Krembil Brain Institute, University Health Network, Toronto, ON, Canada (A. Morozova).

**Keywords:** depression, hypertension, magnetic resonance imaging, neuroimaging, perivascular spaces, stress

## Abstract

**BACKGROUND:**

Brain perivascular spaces (PVS) are emerging magnetic resonance imaging markers of microvascular function and waste metabolite clearance. Although PVS enlargement has been linked to aging and vascular risk, it remains unclear whether PVS morphometry reflects shared familial microvascular characteristics and how these are shaped by individual vascular, physiological, and neuropsychiatric factors. We investigated whether PVS morphometry captures these familial characteristics in addition to individual determinants.

**METHODS:**

We analyzed 1183 participants from the Stratifying Depression and Resilience Longitudinally family-based cohort, including 324 individuals with first-degree relatives. Automated magnetic resonance imaging segmentation quantified PVS volume, count, density, and median length in the centrum semiovale and basal ganglia. Linear mixed-effects models assessed associations with age, hypertension, hair cortisol, depressive symptom scores, and hand grip strength while accounting for familial clustering.

**RESULTS:**

In 1050 individuals (59.5% female; mean age, 59.3±10.1 years), PVS burden increased with age (PVSvolume%ROI, β=0.18 [95% CI, 0.11–0.26]; *P*<0.0001), current depressive symptoms across both regions (PVS density: centrum semiovale, β=0.092 [0.023–0.16]; *P*=0.009; BG, β=0.11 [0.043–0.18]; *P*=0.002; largely robust to false discovery rate correction), and with higher hair cortisol (PVS count β=0.08 [0.003–0.15]; *P*=0.041; borderline after false discovery rate correction) and weaker grip strength (PVSvolume%ROI β=−0.09 [−0.16 to −0.02]; *P*=0.013), in the centrum semiovale. Familial clustering was significant for PVS volume (β=0.22 [0.096–0.52]; *P*=0.013) and median length (β=0.28 [0.16−0.49]; *P*=0.0003), independent of other factors.

**CONCLUSIONS:**

PVS morphometry reflects shared familial microvascular phenotypes and neuropsychiatric influences beyond hypertension, highlighting both familial and individual determinants of PVS burden and morphology. These findings support PVS morphometry as a neuroimaging marker of cerebral microvascular health.

Brain perivascular spaces (PVS) are magnetic resonance imaging (MRI)-visible compartments surrounding cerebral perforating vessels, serving as conduits for waste clearance and maintaining microvascular and brain homeostasis.^1–3^ Enlargement of PVS has been associated with aging, hypertension, and neurovascular and neurodegenerative conditions, including cerebral small vessel disease, multiple sclerosis, and dementia.^4–7^ PVS morphology exhibits high interindividual variability,^2^ with genetics being a substantial predisposing factor,^8,9^ as demonstrated by recent large genome-wide association studies.^10,11^ However, genetic association alone does not fully capture the extent to which microvascular and perivascular structure is shared within families, where inherited factors coexist with shared developmental and environmental influences.

Microvascular function may be modulated by neuropsychiatric and physiological factors, including chronic stress, depressive symptoms, and physical strength, through overlapping and potentially interacting pathways such as inflammation, oxidative stress, and vascular dysregulation.^12–15^ Chronic stress is a well-established proinflammatory trigger that disrupts microcirculatory function^12^ and is a risk factor for cardiovascular and neurodegenerative disorders,^16^ while depressive symptoms have been linked to neuroinflammation and decreased cerebral blood flow.^16,17^ Reduced muscular strength is associated with increased cardiometabolic risk, cardiovascular and overall mortality, and cognitive decline.^18^ These factors may act in concert to influence small vessel integrity, with PVS morphology reflecting both individual determinants and familial impact.

To date, few studies have examined the relationship between neuropsychiatric factors and PVS. Existing work suggests associations between stress- or trauma-related exposures and increased PVS burden,^19^ potentially mediated by neuroinflammatory processes,^20^ as well as links between PVS burden and poststroke depression.^21^ However, the extent to which neuropsychiatric factors independently influence PVS enlargement and morphometric features remains unclear. Moreover, while the heritability of PVS burden has been demonstrated,^10,11^ and the extent to which PVS morphometry reflects shared familial microvascular architecture has not been established.

In this study, we examined familial and individual determinants of PVS burden and morphology in a large, population-based cohort, including familial clustering of PVS morphometric features among first-degree relatives to distinguish shared familial from individual influences, while evaluating associations with neuropsychiatric factors (chronic stress assessed via hair cortisol and depressive symptoms measured by the Quick Inventory of Depressive Symptomatology^22^) and physical strength (hand grip^23^). Moving beyond conventional visual ratings or single quantitative metrics, we applied a comprehensive computational morphometric approach, quantifying PVS volume, count, density, and median length to capture distinct dimensions of PVS architecture, with volume and density reflecting PVS burden, and count and length reflecting PVS morphology. White matter hyperintensities, established markers of small vessel disease^24^ topologically linked with PVS,^25^ were included as covariates to isolate effects specific to PVS. We hypothesized that familial membership would confer similarity in PVS morphometry, and that age, vascular risk, higher stress, and depressive symptoms would associate with greater PVS burden, whereas stronger hand grip would associate with lower PVS burden as evidence of microvascular health.

## Methods

### Data Availability

The original data used and the data generated, namely, PVS and PVS ROI segmentations and metrics, are held in the Edinburgh DataVault. De-identified data are available to researchers on request to the GS:SFHS (Generation Scotland: Scottish Family Health Study) Access Committee (access@generationscotland.org).

### Sample

This study is reported in accordance with the STROBE guidelines (Strengthening the Reporting of Observational Studies in Epidemiology) for observational studies. The data set comes from the STRADL study (Stratifying Depression and Resilience Longitudinally),^26^ a population-based longitudinal study examining the biological and psychological mechanisms of depression and resilience. Inclusion criteria were participation in the study, availability of MRI data, and availability of relevant demographic and clinical variables required for the present analyses. Participants were excluded if structural MRI data were missing or of insufficient quality for analysis.

### Image Processing

The neuroimaging protocol of the primary study that provided data for this analysis is published.^26^ We used previously defaced T1-weighted, T2-weighted and fluid attenuated inversion recovery MRI, as well as the brain parcellation done by Freesurfer version 6.0 (https://surfer.nmr.mgh.harvard.edu/), all provided by the Generation Scotland Data Access Committee (https://genscot.ed.ac.uk/for-researchers/access), together with the rest of the variables used in the analyses. We coregistered all MRI sequences to the nearly isotropic (1×0.94×0.94 mm^3^) T1-weighted space, where the Freesurfer segmentations are, and obtained the intracranial volume using FLIRT (FMRIB’s Linear Image Registration Tool)^27^ and BET2 (Brain Extraction Tool 2).^28^ We applied Gaussian clustering to the multisequence (4D) volume obtained by concatenating the 3 coregistered MRI sequences to extract the brain tissue separately from the pure CSF, the meningeal layers and main venous pathways. We obtained initial priors of the basal ganglia (BG) and white matter regions (mainly centrum semiovale [CSO], excluding the internal and external capsules that were included in the BG region) from Freesurfer segmentations. We refined them to ensure that venous pathways, membranes and CSF were not included, by applying the masks generated from the multispectral Gaussian clustering segmentations. Then, we mapped our (3D) regions of interest (ROIs) to the T2-weighted space, of higher spatial resolution (ie, 0.5×0.48×0.48 mm^3^), where we performed the PVS segmentations using the thresholded output from the Frangi filter as described in Ballerini et al.^29^ From the PVS segmentations, we calculated the percentage volume and the density of PVS in each ROI (PVS counts in a ROI volume) and determined the average PVS length per ROI using the Bezier curves approximation.^30^ White matter hyperintensities (WMH) were segmented automatically using nn-UNet, a self-configuring U-shaped neural network for segmenting biomedical images using deep-learning,^31^ previously trained on a set of 162 fluid attenuated inversion recovery MRI data from individuals from studies of aging and patients with mild stroke, all with different degrees of small vessel disease.^32^ The out-of-the-box nn-UNet pretrained model used is 3D with a batch size of 2 and a patch size of 112×160×128 voxels. It uses as input fluid attenuated inversion recovery images, resampled to 0.94×0.94×0.90 mm^3^ voxel-size resolution.

### Overview of Methods for Glucocorticoid Extraction/Grip Strength/QIDS/Hypertension

Demographic data (age and sex) were obtained within the GS:SFHS^33^ between 2006 and 2011. Grip strength, hair sample, and Quick Inventory of Depressive Symptomatology (QIDS) were obtained at the same time as the MRI in an in-person assessment wave in 2019 for the STRADL study (Figure S1). Blood pressure was measured during the same in-person clinical assessment, with 3 readings obtained at 1-minute intervals using an appropriately sized cuff after a 5-minute seated rest. The mean of the second and third readings was used to determine blood pressure. Hypertension was defined using the following criteria: systolic blood pressure ≥140 mm Hg, diastolic blood pressure ≥90 mm Hg, or current use of antihypertensive medication. Participants meeting any of these criteria were coded as “yes”; all others were coded as “no.” For the present analyses, the derived binary hypertension variable (yes/no) provided within the data set was used. Grip strength was determined using a Patterson Medical Jamar hand dynamometer. A small sample (ie, ≈3 mm diameter) of hair was collected from the posterior vertex region of the head for extracting cumulative cortisol using organic solvents in pulverized hair and processed using liquid chromatography-mass spectroscopy.^26^

### Statistical Analysis

We applied a linear mixed effects model to account for possible correlations in the PVS characteristics among first-degree relatives. Family ID was included as a random intercept to model shared variance within families. Age, hypertension, hair cortisol, grip strength, WMH burden and QIDS score were entered as fixed effects to examine their influence on the PVS metrics. Sex was considered as a covariate in preliminary analyses, but it was not included in the main models because it was not statistically significant across most outcomes (Table S1). This model enabled to assess both within-family and between-family variation, including comparisons between family members and unrelated individuals, providing insight into the contribution of familial factors on the outcome. Due to skewed distributions, WMH burden and hair cortisol concentration were log-transformed before inclusion in the models. We computed 8 models—4 per brain region (CSO and BG)—including PVS volume as the primary, hypothesis-driven measure of PVS burden, and PVS density, count, and median length as exploratory morphometric outcomes, since these metrics have not yet been widely assessed in previous studies.

A sensitivity analysis excluding participants with a prior stroke was conducted to assess the robustness of findings (Table S2). Since the data set contained both individuals and families, we also performed a sensitivity analysis by repeating the analysis on a sample that excluded individuals without family members (ie, restricting the sample to families with at least 2 members). Family ID was added as a random intercept, as in the primary model, while age was added as a random slope to assess potential differences in its effect across families. We did not exclude participants with a history of stroke or any other incidental finding in their brain scans.

To assess the potential impact of multiple comparisons, we applied a false discovery rate (FDR) correction as a sensitivity analysis for each brain region using the *q* value method, and q-values<0.05 were considered statistically significant (Table S3). All statistical analyses were performed using the Stata software package (version 18, Stata Corp, 2023. Stata Statistical Software: Release 18. College Station, TX: Stata Corp LLC).

## Results

### Sample Characteristics

The flow chart of the recruitment and final sample used in this study is shown in Figure S1. Briefly, from the 21 525 members of the GS:SFHS cohort^32^ eligible for re-contact, 5649 were invited to participate in STRADL. From them, 646 declined, and 3358 did not respond. From those who consented to participate, we considered for this study 1183 subjects that had all relevant clinical and demographic data available, with MRI assessment performed at 2 locations, Aberdeen (51%) and Dundee (49%), in Scotland, United Kingdom, using 3T MRI scanners. From them, 859 individuals were unrelated, and 324 were part of 136 families of between 2 and 6 members—all first-degree relatives. It is worth noting that 128 had missing MRI data (ie, either an incomplete MRI scan or missing or corrupted MRI sequences). Therefore, we used MRI data from 1055 study participants. After image analysis quality control, 1050 measurements were included in the statistical analyses.

### Descriptive Statistics

The derivation of the study sample (n=1050) from the initial cohort (n=1183) is shown in Figure S1, with exclusions largely due to unavailable MRI data (n=103) or missing MRI sequences required for analysis (n=25). Demographic, clinical, and neuroimaging characteristics are presented in Table 1. No statistically significant differences in the average parameters evaluated were observed between the sample included and the total sample comprised of those who consented and underwent the relevant clinical tests for this study (see values in Table 1). The distribution of family sizes is summarized in Table 2. Each family size represents the number of first-degree members per family. The mean age was 59.3±10.1 years, and 625/1050 (59.5%) were female. Most did not have hypertension or prior stroke.

**Table 1.**
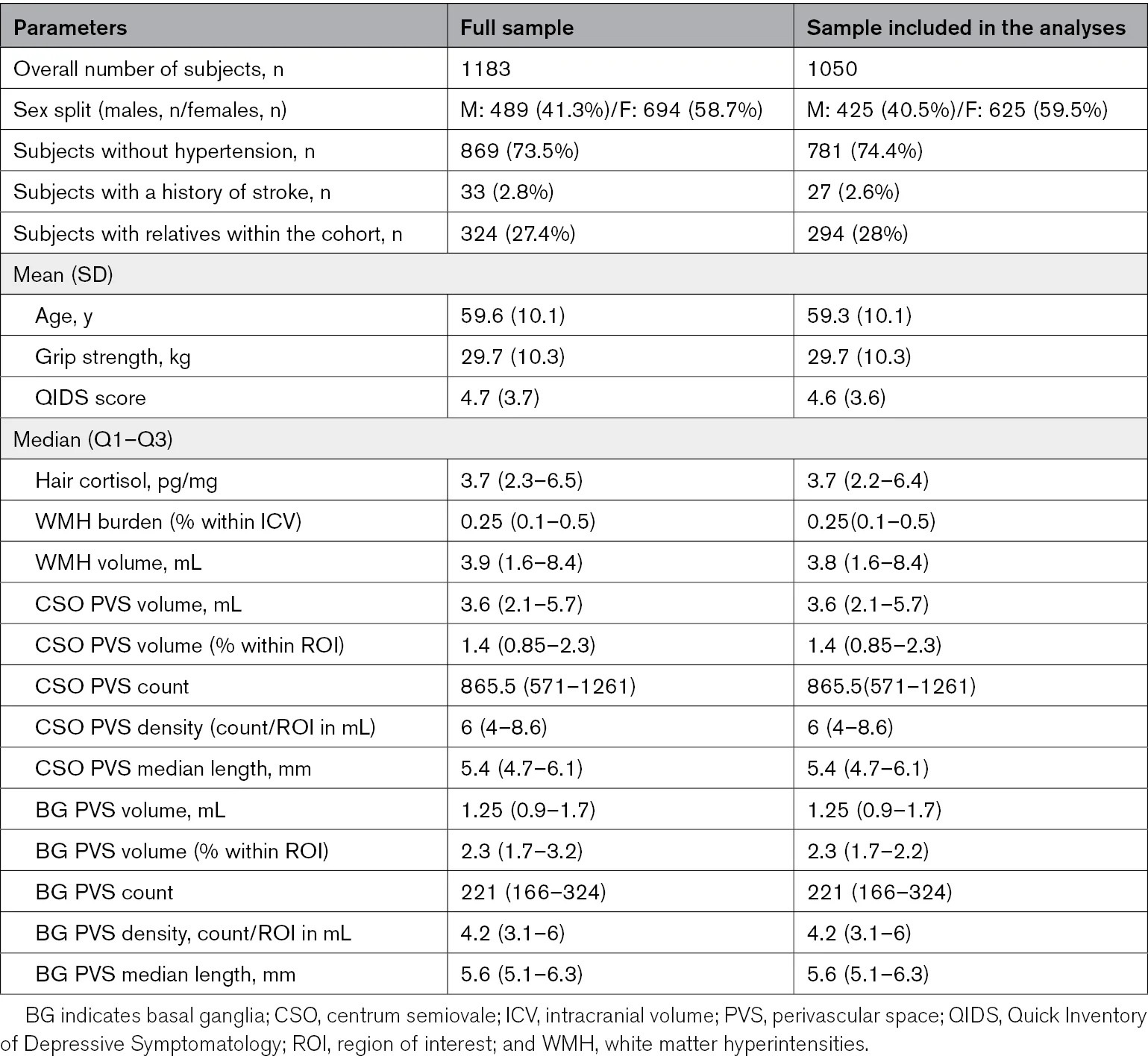
Study Data Overview

**Table 2.**
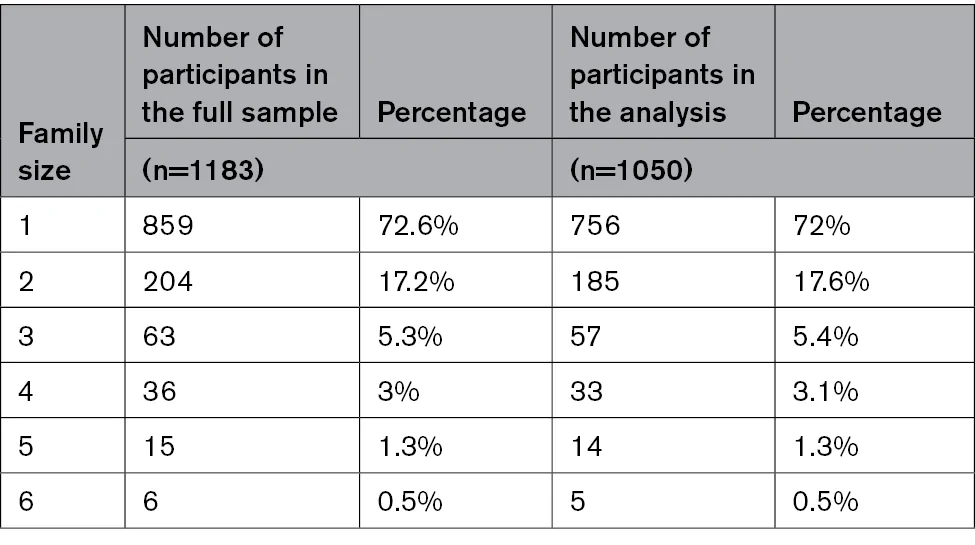
Distribution of Family Cluster Sizes in the Sample (First-Degree Relatives Only)

### Primary Analyses

**Figure 1.**
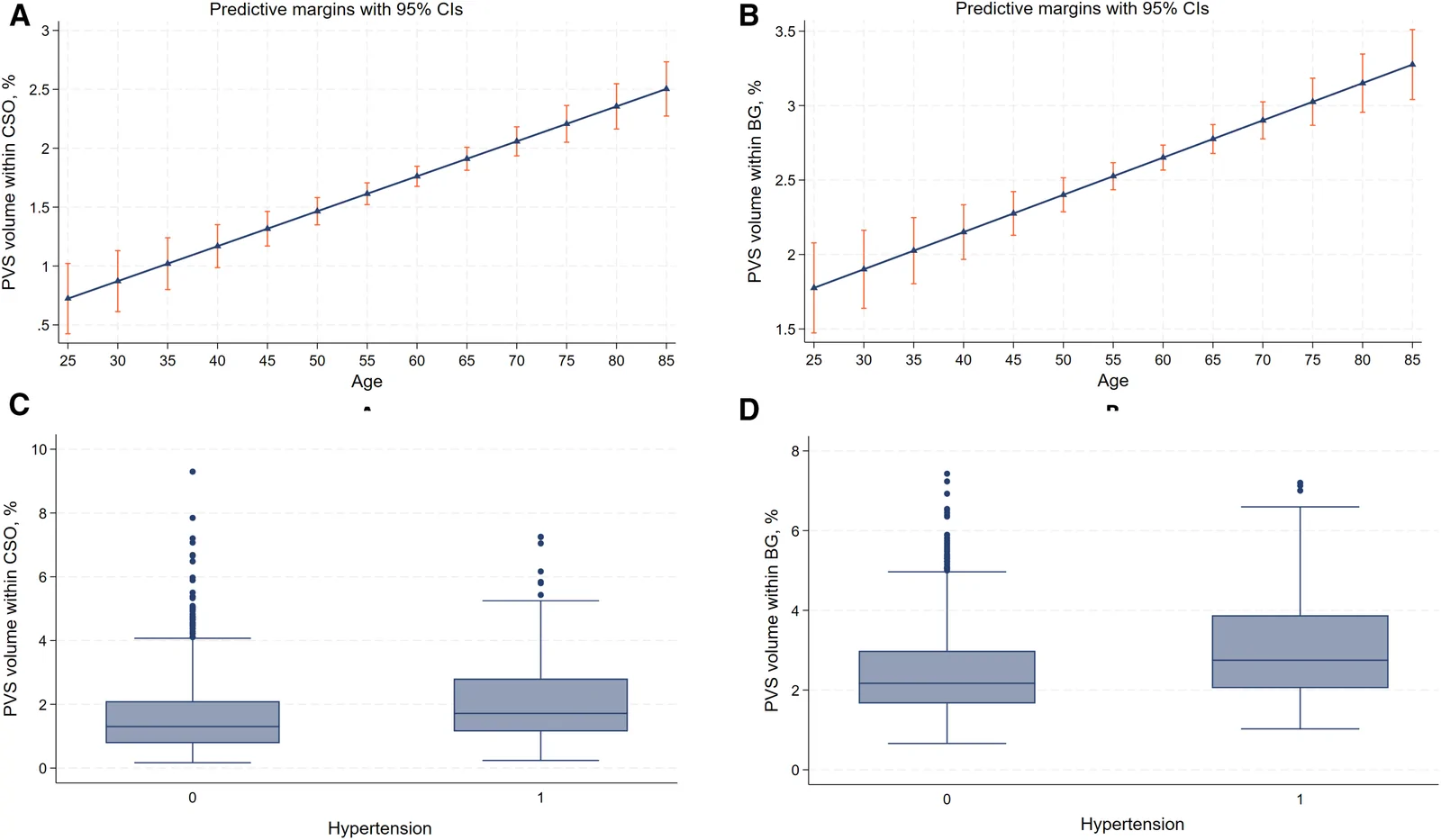
**Associations of perivascular spaces (PVS) volume with age and hypertension. A** and **B**, Association between age (in years) and PVS volume (as % of the region of interest) in the centrum semiovale (CSO; **A**), and basal ganglia (BG, **B**). **C** and **D**, Association between hypertension (yes=1 and no=0) and PVS volume (as %in the region of interest) in the CSO (**C**), and BG (**D**). For age (**A**) and (**B**), effect sizes, representing the predicted change in PVS volume per year, are indicated on the *y* axis.

**Figure 2.**
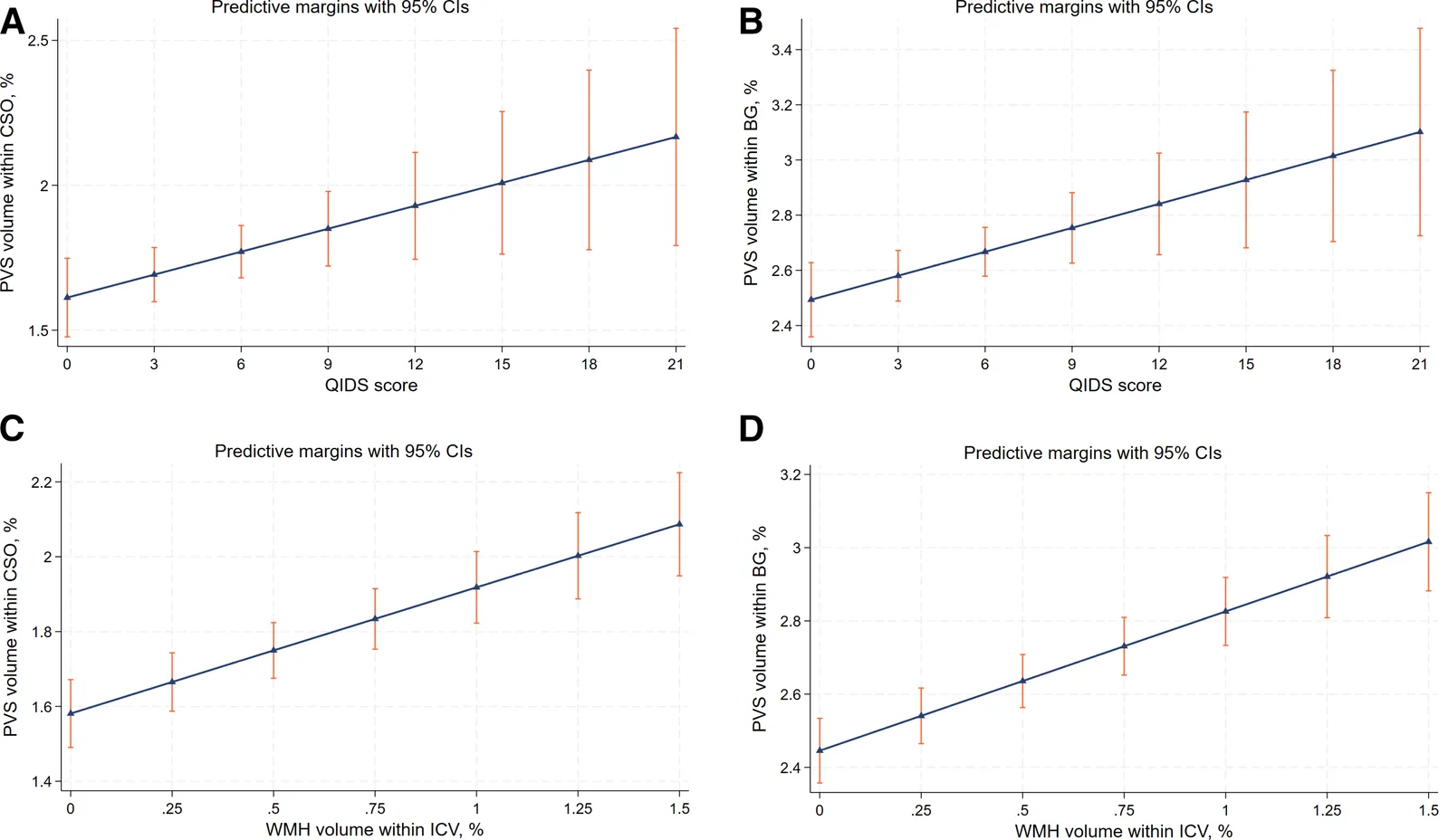
**Associations of perivascular spaces (PVS) volume with depressive symptoms and white matter hyperintensities. A** and **B**, Association between the Quick Inventory of Depressive Symptomatology (QIDS) score and PVS volume (as % of the region of interest) in the centrum semiovale (CSO; **A**), and basal ganglia (BG; **B**). **C** and **D**, Association between white matter hyperintensities (WMH) volume within the intracranial volume (ICV) in % and PVS volume (as % in the region of interest) in the CSO (**C**), and BG (**D**). WMH volume was truncated at the 95th percentile for visualization purposes only. Effect sizes, representing the predicted change in PVS volume per unit increase in the respective predictor, are indicated on the *y* axis to provide an estimate of the magnitude of the associations.

A linear mixed effects model was built for each PVS measurement (PVS volume % in the ROI, count, density, and median length) as an outcome in both the CSO and the BG. The results from all models are summarized in Table 3 (standardized values) and in Table S4 (nonstandardized values). The stated β-coefficients are standardized with 95% CIs in square brackets unless otherwise stated. Associations between PVS volume (% in ROIs; the PVS characteristic representative of the PVS burden) and statistically significant covariates are shown in Figures 1 and 2. The variance component for Family ID was significant in the CSO with the percentage of PVS volume in this region as the outcome (standardized β=0.22 [95% CI, 0.096–0.52]; *P*=0.013), and PVS median length (β=0.28 [0.16–0.49]; *P*=0.0003), indicating that a substantial portion of the variance in CSO is explained by familial factors. In the BG, the family ID effect was not associated with PVS volume%ROI.

**Table 3.**
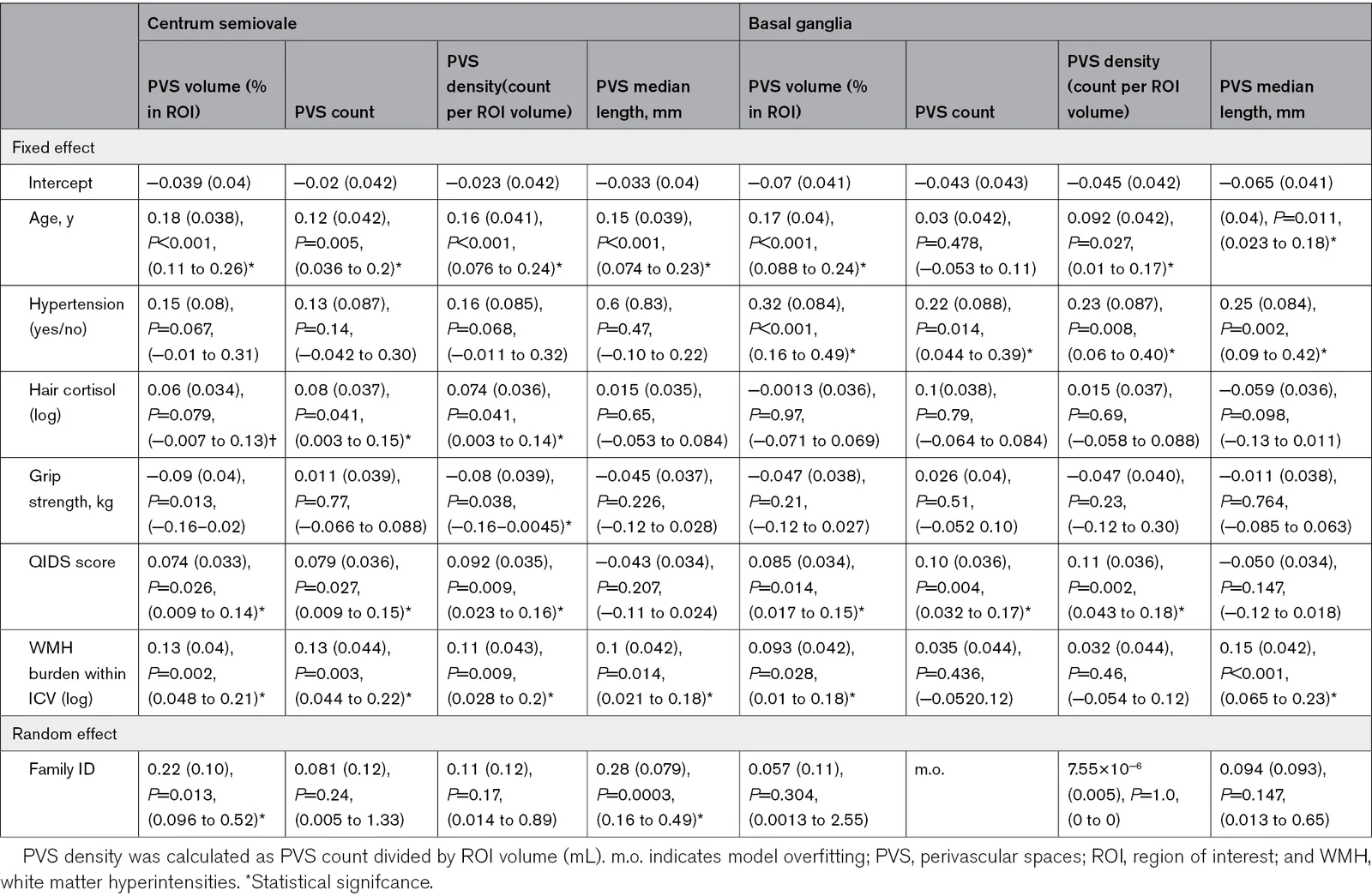
Linear Mixed Effects Models—Standardized Results (β, SE, *P* Values, 95% CI)

Age was a highly significant predictor of the PVS burden in the CSO across all models for PVS volume%ROI (β=0.18 [0.11–0.26]; *P*<0.0001), density (β=0.16 [0.076–0.24]; *P*<0.0001), median length (β=0.15 [0.074–0.23]; *P*<0.0001), and count (β=0.12 [0.036–0.2]; *P*=0.005); Figure 1A; Table 3; Table S4. In the BG, age was associated with PVS volume%ROI (β=0.17 [0.088–0.24]; *P*<0.001); Figure 1B, PVS density (β=0.092 [0.01–0.17]; *P*=0.027), and PVS median length (β=0.1 [0.023–0.18]; *P*=0.011; Table 3).

Hypertension was not associated with PVS measurements in the CSO apart from a trend for PVS volume%ROI (β=0.15 [−0.01 to 0.31]; *P*=0.067; Figure 1C), but was associated with all PVS measures in the BG:PVS volume%ROI (β=0.32 [0.16–0.49]; *P*<0.001; Figure 1D); PVS count (β=0.22 [0.044−0.39]; *P*=0.014); PVS density (β=0.23 [0.06−0.40]; *P*=0.008); and PVS median length (β=0.25 [0.09−0.42]; *P*=0.002); Table 3).

The associations of the QIDS depression score with PVS were the same in the CSO and the BG: PVS volume%ROI (CSO, β=0.07 [0.009−0.14]; *P*=0.026); Figure 2A; BG, β=0.085 [0.017−0.15]; Figure 2B); PVS count (CSO, β=0.079 [0.009−0.15]; *P*=0.027, BG, β=0.1 [0.032−0.17]; *P*=0.004); PVS density (CSO, β=0.092 [0.023−0.16]; *P*=0.009; BG, β=0.11 [0.043−0.18]; *P*=0.002). There were no significant associations between the QIDS score and PVS median length in either of the brain regions.

WMH volume (% within ICV) was associated with PVS burden in the CSO across all models: PVS volume%ROI (β=0.13 [0.048–0.21]; *P*=0.002; Figure 2C), PVS count (β=0.13 [0.044–0.22]; *P*=0.003), PVS density (β=0.11 [0.028–0.2]; *P*=0.009), and PVS median length (β=0.1 [0.021–0.18]; *P*=0.014). In the BG, WMH volume (% within ICV) was associated with the PVS volume%ROI (β=0.093 [0.01–0.18]; *P*=0.028; Figure 2D) and PVS median length (β=0.15 [0.065–0.23]; *P*<0.001).

**Figure 3.**
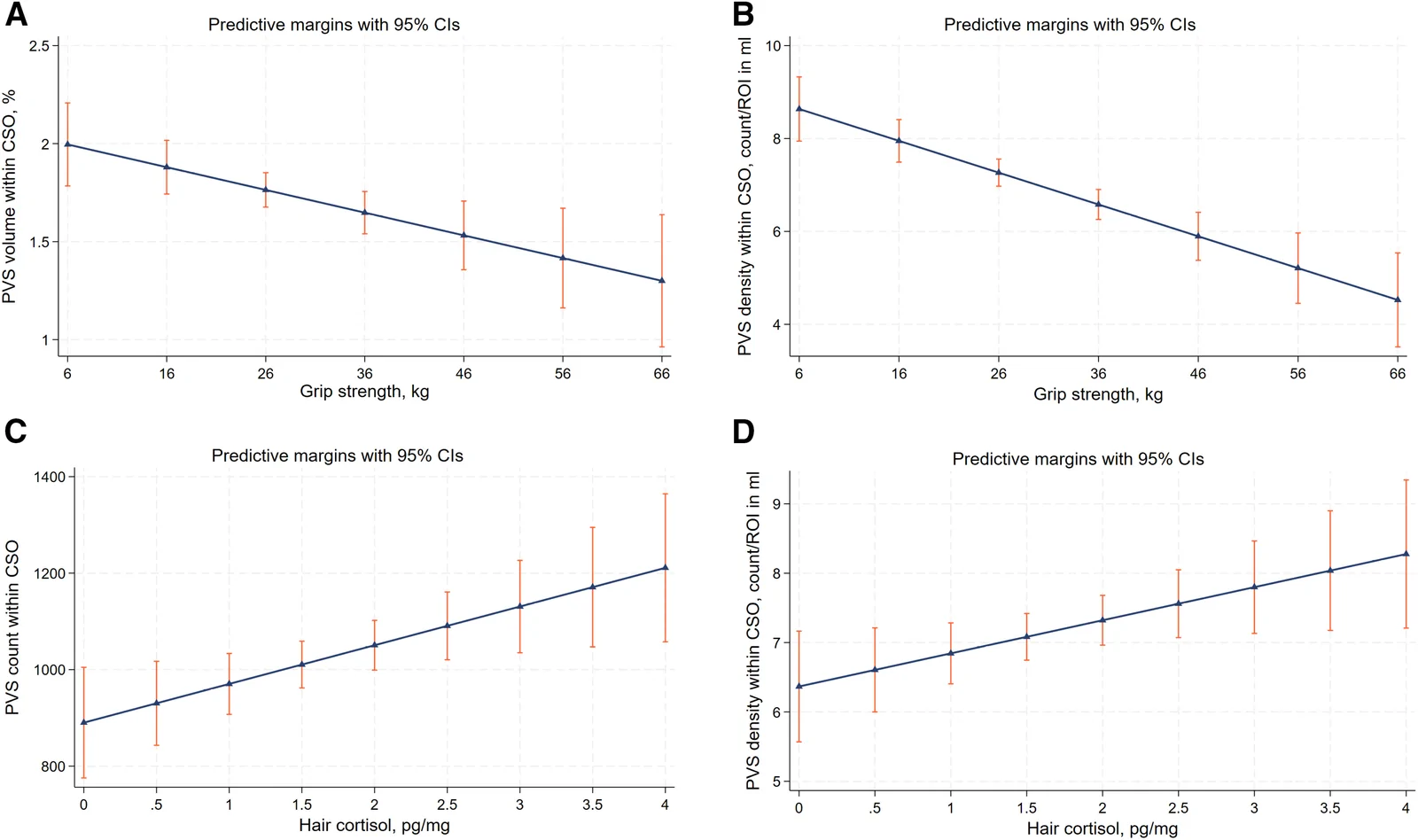
**Associations between perivascular spaces (PVS) measures in the centrum semiovale (CSO) and grip strength and hair cortisol. A** and **B**, Associations of grip strength (kg) with PVS volume (as % of the region of interest [ROI]) and PVS density (count per ROI volume, mL). **C** and **D**, Associations of hair cortisol (pg/mg, log-transformed) with PVS count and PVS density (count per ROI volume, mL). Effect sizes, representing the predicted change in PVS volume per unit increase in the respective predictor, are indicated on the *y* axis to provide an estimate of the magnitude of the associations.

Grip strength was negatively associated with increased percentage of PVS volume%ROI (β=−0.09 [−0.16 to −0.02]; *P*=0.013; Figure 3A) and PVS density (β=−0.08 [−0.16 to −0.0045]; *P*=0.038; Figure 3B) in the CSO. It was not associated with any PVS measurements in the BG.

Hair cortisol level was associated with 2 CSO PVS measurements: PVS count (β=0.08 [0.003−0.15]; *P*=0.041; Figure 3C) and PVS density (β=0.074 [0.003−0.14]; *P*=0.041; Figure 3D). Association with CSO PVS volume (as % in the CSO ROI) was of borderline significance (β=0.06 [−0.007 to 0.13]; *P*=0.079). There were no associations between hair cortisol and the BG PVS measurements.

### Sensitivity Analyses

Excluding participants with a prior stroke (n=27) did not substantially alter the direction, magnitude, or significance of the observed associations (Table S2).

Including sex as a covariate in the models generally did not result in statistically significant effects for sex across most PVS outcomes (Table S1). An exception was observed for BG median PVS length, where both sex and grip strength showed significant associations. We also tested a sex × grip interaction by including an interaction term in the same model; this was not significant (standardized β=0.008 [95% CI, −0.202 to 0.219]; *P*=0.937), indicating that the association between grip strength and BG PVS length is independent of sex.

**Figure 4.**
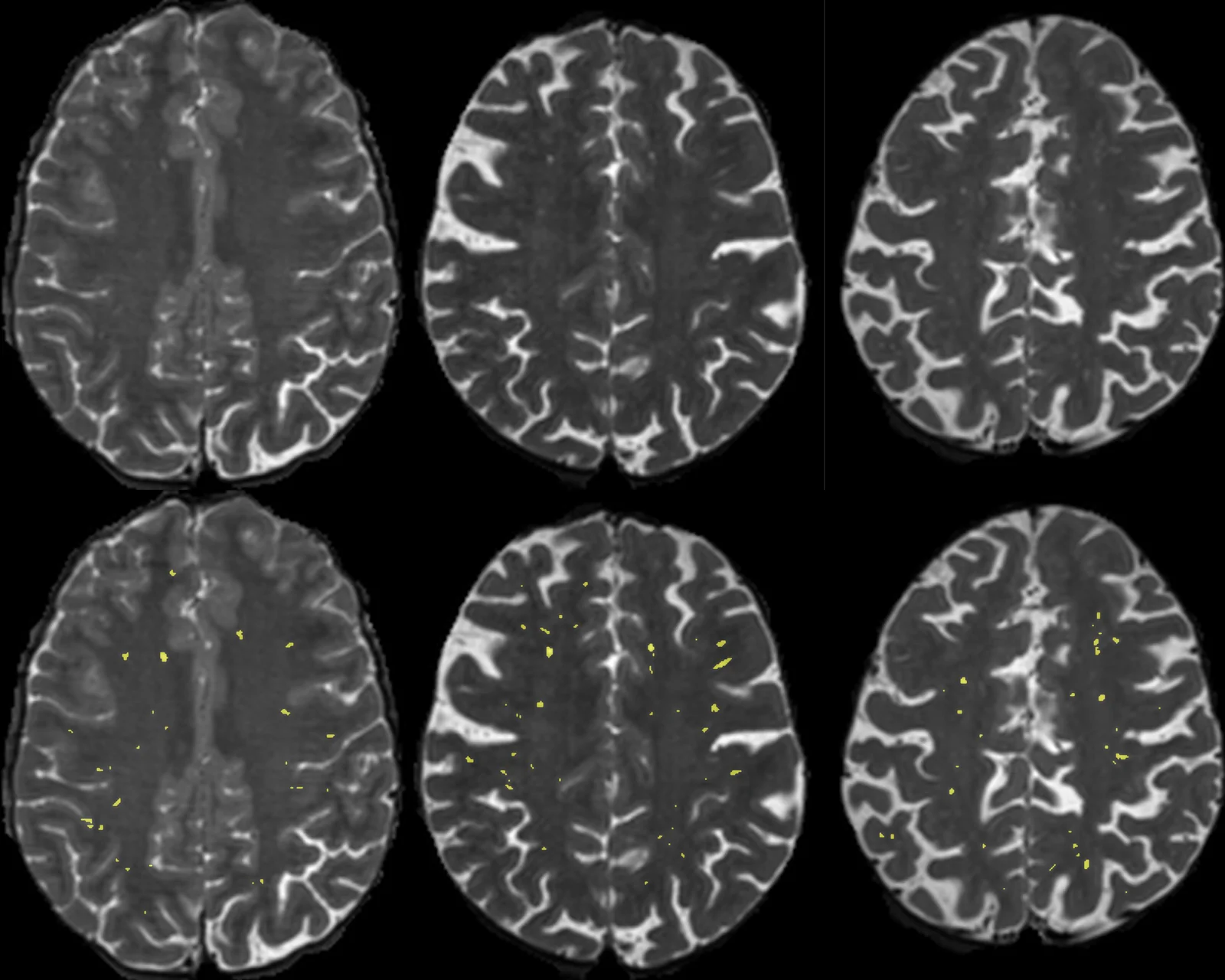
**Equivalent axial T2-weighted magnetic resonance imaging slice in the centrum semiovale, with the perivascular spaces (PVS) segmented (bottom row) in yellow, in 3 consecutive generations of the same family (younger to older: left to right).** Observe a similar number of PVS counted regardless of age differences, and similarities in the topological (ie, spatial) distribution patterns in consecutive generations.

To further evaluate the robustness of familial effects, we repeated the original analyses in the 2 models in which the random effect of family ID was statistically significant (PVS volume%ROIs and PVS median length in the CSO as outcomes; Table 3). Figure 4 shows PVS in the CSO on T2 MRI in 3 generations of the same family. First, we replicated the original model with all covariates but restricted the sample to families with at least 2 members (n=294), excluding individuals without family members. This allowed us to assess whether family ID was associated with the outcomes, specifically, PVS volume and PVS median length in the CSO. The random effect of family ID remained significant for both PVS outcomes (PVS volume%ROI: variance=0.21, likelihood-ratio (LR) test=4.14, *P*=0.021; PVS median length: variance=0.44, LR test=10.49, *P*=0.0006), indicating persistent familial clustering in the restricted sample. Fixed-effect estimates remained broadly consistent in direction and magnitude across models, although only age remained statistically significant in this restricted sample, likely due to reduced power.

Next, we repeated the 2 analyses on the restricted sample, adding age as a random slope to the model to examine whether the effect of age on the outcomes varied across families. The model, including a random slope for age, showed significant random effects, indicating heterogeneity in age-related effects across families (PVS volume%ROI: age variance=0.000093, LR test χ^2^(2)=8.53, *P*=0.014; PVS median length: age variance=0.00013; LR test χ^2^(2)=13.18; *P*=0.0014). Model fit was similar across models in the restricted sample (Akaike Information Criterion PVS volume%ROI, 669.95 versus 667.56; PVS median length Akaike Information Criterion, 695.7 versus 693.01), with the model including age as a random slope showing slightly better fit.

To address the potential for inflated type I error due to multiple comparisons, we performed an FDR correction for each brain region. The FDR-adjusted q-values largely confirmed the main findings, with 12 out of 17 associations staying significant in the CSO, and 10 out of 12 associations keeping their significance in the BG; the rest of the values became borderline (*q*=0.054–0.068) as a result. Full FDR-adjusted *q* values are provided in Table S3.

## Discussion

Our study demonstrates that PVS morphometry is shaped by familial, neuropsychiatric, and physiological factors, as well as hypertension, providing novel insight into cerebral microvascular structure and function. Evidence of familial clustering in CSO PVS volume and median length highlights shared microvascular phenotypes within families, encompassing genetic, epigenetic, socio-economic, and early life environmental influences. Hypertension, neuropsychiatric, and physiological factors were additionally associated with PVS features independently of familial effects, while regional dissociation between CSO and BG PVS suggests distinct underlying pathophysiology and differential relevance to small vessel disease phenotypes. Together, these results underscore the multifactorial determinants of cerebral microvasculature and support PVS morphometry as a sensitive neuroimaging marker of microvascular function.

### Familial Influences

Significant familial clustering was observed for CSO PVS volume and median length, independent of individual determinants (Figure 4). These findings indicate that aspects of perivascular morphometry may be shared within families, reflecting influences that extend beyond individual-level vascular risk factors. Prior genetic studies have reported heritable contributions to vascular morphology.^10,11,34,35^ The present findings reflect familial clustering rather than heritability per se. Such familial patterns likely arise from a combination of influences shaping microvascular architecture across generations, including genetic factors, epigenetic processes, and shared environmental exposures such as early life conditions, socioeconomic context, and intergenerational vascular risk profiles. While age-related perivascular changes are universally observed, our models indicate that their trajectories vary across families, likely reflecting familial vascular phenotypes associated with susceptibility to microvascular remodeling.

### Age and Hypertension

Age and hypertension were strongly associated with PVS measures, aligning with previous reports.^1,4^ Age effects were consistent across CSO and BG measures, except for BG PVS count, likely reflecting the lower absolute PVS numbers in this region. Hypertension showed stronger associations in the BG and weaker effects in the CSO. Overall, pathological vascular changes are likely not restricted to the BG but may first be apparent in its small vessels, which perhaps are most proximate to systemic factors like blood pressure. In contrast, the CSO penetrating arterioles are further from the systemic vasculature and have different perivascular membranes, so they may be less sensitive to systemic effects. The binary nature of our hypertension variable may limit sensitivity, as cumulative blood pressure better captures continuous vascular impact.^36^

### Neuropsychiatric Factors

Chronic stress, measured via hair cortisol, was associated with increased CSO PVS count and density, although this association was borderline after correction for multiple comparisons. Cortisol predominantly affects cortical regions, promoting neuroinflammation, which may, in turn, impair perivascular clearance.^19,20,37^ Depressive symptoms, assessed via QIDS at the time of MRI, were associated with higher PVS volume, count, and density in both regions. These associations may reflect convergent influences of stress and depression on microvascular and perivascular function, potentially involving neuroinflammatory or metabolic pathways.^38,39^ Using continuous measures at the time of MRI likely captured subtle associations beyond those identifiable using categorical yes/no depression diagnoses, given that depression is often under-reported.^40^ Given the cross-sectional design, we cannot infer causality or directionality, and residual confounding by sleep, metabolic health, or systemic inflammation remains possible.

### Physical Strength

Hand grip strength inversely correlated with CSO PVS volume and density (reflecting PVS burden), indicating that better physical fitness may preserve microvascular health. Hand grip is a validated marker of cardiovascular, cognitive, and general health,^41–43^ supporting its potential as a simple proxy for perivascular function. Interestingly, the primary motor and sensory white matter pathways make up a prominent proportion of the CSO as they fan out to the cortex, whereas the same pathways are tightly bunched, taking up a smaller proportion of the BG region. Perhaps this is why a PVS burden-grip strength association was detected in the CSO rather than the BG. In contrast, adjustment for sex revealed a positive association between BG PVS median length and grip strength, opposite in direction to the associations observed for CSO PVS burden measures. As this association was specific to PVS morphology and emerged only after adjustment for sex, it may reflect regional sex differences in vascular organization within deep perforating arteries of the BG region rather than a pathological clearance mechanism. Given the isolated nature of this finding, cautious interpretation is warranted.

### Regional Differences and WMH

WMH burden correlated with PVS measures, consistent with some prior studies.^44–47^ Adjusting for WMH allowed isolation of PVS-specific associations, consistent with PVS morphological changes reflecting early microvascular changes preceding overt WMH.^48,49^

Regional differences between BG and CSO likely reflect anatomic and functional distinctions, shaping region-specific PVS susceptibility to vascular and physiological stress. BG PVS appear to be linked to hypertensive arteriopathy affecting deep perforating vessels, consistent with the significant associations between hypertension and BG PVS observed across our models. In contrast, CSO PVS are thought to reflect periarteriolar and glymphatic processes within cortical and subcortical white matter, capturing broader microvascular and perivascular dynamics related to cortical vascular organization and fluid clearance, in line with recent evidence of the distinctive mechanistic dissociation between these regions.^50^

In the present study, familial clustering was observed for CSO but not BG PVS, suggesting that familial microvascular phenotypes may be more strongly expressed in cortical perivascular architecture, whereas perivascular changes around deep perforating vessels may be more strongly influenced by individual-level vascular risk factors, namely hypertension. Overall, neuropsychiatric and physiological factors likely act in concert with vascular influences, alongside familial characteristics, to shape PVS morphometry. While familial clustering establishes baseline microvascular patterns, individual determinants such as stress, depressive symptoms, hypertension, and physical strength are additionally associated with PVS morphology and burden. This emphasizes that both familial and nonfamilial factors converge to determine cerebral microvascular structure.

### Limitations

The cross-sectional design precludes causal inference. The full analytic sample represents STRADL participants with complete MRI and clinical data, while familial clustering was estimated from participants with at least 1 first-degree relative. As the family component is a more selective group, these analyses may not fully reflect the broader population-based STRADL cohort, which also includes participants without relatives, potentially limiting generalizability. Hypertension was modelled as a binary variable, which does not capture blood pressure severity, variability, or duration of exposure. Consequently, this approach may underestimate cumulative vascular exposure and obscure potential dose–response relationships with microvascular outcomes, particularly in familial analyses where microvascular traits may cluster within families. Given the large number of statistical tests conducted, there is a potential for inflated type I error; we therefore additionally performed an FDR correction per brain region, which largely confirmed the main findings while identifying a few borderline associations, warranting cautious interpretation. Only 8.8% of participants had a formal depression diagnosis, but continuous QIDS scores at MRI provided robust measures of symptom severity. Longitudinal studies are needed to better characterize PVS dynamics and familial influences over time.

### Conclusions

PVS morphometry reflects shared familial microvascular architecture and is associated with hypertension, neuropsychiatric, and physiological factors. These findings highlight both familial and individual contributions, demonstrate regional differences in PVS morphology and burden, and support PVS morphometry as a noninvasive neuroimaging marker of cerebral microvascular function.

## Article Information

### Acknowledgments

The authors would like to thank all of the Generation Scotland participants for their contribution to this study. The authors also thank the research assistants, clinicians, and technicians for their help in collecting the data, and Generation Scotland administration for making it available for research.

### Disclosures

Dr Wardlaw reports grants from Medical Research Council; grants from Wellcome Trust; compensation from Research Councils UK for other services; grants from Mrs Gladys Row Fogo Charitable Trust; grants from Alzheimer’s Research UK; grants from Weston Brain Institute; and grants from Alzheimer’s Society. The other authors report no conflicts.

### Supplemental Material

Tables S1–S4

Figure S1

STROBE Checklist

## Supplementary Material


