# Supplementary material for: Brain Perivascular Space Morphometry Reflects Shared Familial Microvascular Architecture and Neuropsychiatric Influences
Source: Stroke. 2026 Jul 17;57(9):2768–79. doi: 10.1161/STROKEAHA.126.055378 (PMC13506202; doi:10.1161/STROKEAHA.126.055378)
Supplement: Supplementary file 1 [file str-57-2768-s001.pdf]

Brain Perivascular Space Morphometry Reflects Shared Familial Microvascular Architecture and Neuropsychiatric Influences

Alexandra Morozova, Maria del C. Valdés Hernández, Roberto Duarte Coello, Heather Whalley, Andrew McIntosh, Anca-Larisa Sandu Giuraniuc, Gordon D. Waiter, Christopher J McNeil, Douglas Steele, Jennifer A. Macfarlane, Alison Murray, Joanna M. Wardlaw

SUPPLEMENTAL MATERIAL

Table S1. Linear mixed effects models including sex - standardized results (β, standard error (SE), p-values, 95% CI)

	Centrum Semiovale				*Basal Ganglia*			
	**PVS volume (% in ROI)**	**PVS count**	**PVS density (count per ROI volume (in mm³))**	**PVS median length (mm)**	**PVS volume (% in ROI)**	**PVS count**	**PVS density (count per ROI volume (in mm³))**	**PVS median length (mm)**
Fixed Effect								
**Intercept**	-0.07 (0.19)	0.25 (0.20)	-0.02 (0.20)	-0.26 (0.19)	-0.23 (0.19)	0.16 (0.21)	0.009 (0.20)	-0.67 (0.19)
**Sex**	0.02 (0.11), p=0.86, [-0.2 0.24]	-0.16 (0.12), p = 0.18, [-0.40 0.07]	-0.003 (0.12), p = 0.98, [-0.24 0.23]	0.14 (0.12), p = 0.24, [-0.09 0.36]	0.10 (0.12), p = 0.41, [-0.13 0.33]	-0.12 (0.12), p = 0.32, [-0.37 0.12]	-0.03 (0.12), p = 0.79, [-0.27 0.21]	**0.37 (0.12), p = 0.001, [0.15 0.6]**
**Age (years)**	**0.19 (0.04), p<0.001, [0.11 0.26]**	**0.11 (0.04), p = 0.011, [0.02 0.19]**	**0.16 (0.04), p < 0.001, [0.07 0.24]**	**0.16 (0.04), p < 0.001, [0.08 0.24]**	**0.17 (0.04), p < 0.001, [0.09 0.25]**	0.02 (0.04), p = 0.6, [-0.06 0.11]	**0.09 (0.04), p = 0.033, [0.007 0.17]**	**0.12 (0.04), p = 0.002, [0.04 0.20]**
**Hypertension (yes/no)**	0.15 (0.08), p = 0.066, [-0.01 0.31]	0.12 (0.09), p = 0.18, [-0.05 0.29]	0.16 (0.09), p = 0.069, [-0.01 0.32]	0.07 (0.08), p = 0.41, [-0.09 0.23]	**0.33 (0.08), p < 0.001, [0.17 0.50]**	**0.21 (0.09), p = 0.018, [0.04 0.38]**	**0.23 (0.09), p = 0.009, [0.06 0.40]**	**0.28 (0.08), p = 0.001, [0.11 0.44]**

**Hair cortisol (log)**	0.06 (0.03), p = 0.079, [-0.01 0.13]	0.07 (0.04), p = 0.079, [-0.008 0.14]	**0.07 (0.04), p = 0.046, [0.001 0.15]**	0.02 (0.04), p = 0.51, [-0.05 0.09]	0.004 (0.04), p = 0.91, [-0.07 0.08]	0.003 (0.04), p = 0.94, [-0.07 0.08]	0.01 (0.04), p = 0.73, [-0.06 0.09]	-0.04 (0.04), p = 0.3, [-0.11 0.03]
**Grip strength (kg)**	-0.08 (0.06), p = 0.14, [-0.19 0.03]	-0.05 (0.06), p = 0.40, [-0.17 0.07]	-0.08 (0.06), p = 0.17, [-0.20 0.04]	0.01 (0.06), p = 0.91, [-0.11 0.12]	-0.01 (0.06), p = 0.85, [-0.12 0.10]	-0.02 (0.06), p = 0.74, [-0.14 0.10]	-0.06 (0.06), p = 0.32, [-0.18 0.06]	**0.13 (0.06), p = 0.025, [0.02 0.24]**
**QIDS score**	**0.07 (0.03), p = 0.026, [0.009 0.14]**	**0.075 (0.036), p = 0.036, [0.005 0.145]**	**0.09 (0.04), p = 0.009, [0.02 0.16]**	-0.04 (0.03), p = 0.25, [-0.11 0.03]	**0.09 (0.03), p = 0.012, [0.02 0.15]**	**0.10 (0.04), p = 0.006, [0.03 0.17]**	**0.11 (0.04), p = 0.002, [0.04 0.18]**	-0.04 (0.03), p = 0.23, [-0.11 0.03]
**WMH burden within ICV (log)**	**0.13 (0.04), p=0.002, [0.05 0.21]**	**0.12 (0.04), p = 0.006, [0.03 0.21]**	**0.11 (0.04), p = 0.010, [0.03 0.20]**	**0.11 (0.04), p = 0.009, [0.03 0.19]**	**0.10 (0.04), p = 0.022, [0.01 0.18]**	0.03 (0.04), p = 0.53, [-0.06 0.12]	0.03 (0.04), p = 0.49, [-0.06 0.12]	**0.17 (0.04), p < 0.001, [0.08 0.25]**
Random Effect								
**Family ID**	**0.22 (0.1), p=0.013, [0.096 0.52]**	0.078 (0.12), p=0.254, [0.004 1.55]	0.11 (0.12), p=0.17, [0.01 0.89]	**0.28 (0.08), p=0.0004, [0.16 0.49]**	0.06 (0.11), p = 0.3, [0.001 2.47]	m.o.	6.8E-7 (0.001), p=1.0, [0 0]	0.096 (0.095), p=0.15, [0.014 0.67]

Legend: PVS: MRI-visible enlarged perivascular spaces, ROI: Region Of Interest, m.o.: model overfitting

Table S2. Linear mixed effects models excluding subjects with prior history of stroke - standardized results (β, standard error (SE), p-values, 95% CI)

	Centrum Semiovale				*Basal Ganglia*			
	**PVS volume (% in ROI)**	**PVS count**	**PVS density (count per ROI volume (in mm³))**	**PVS median length (mm)**	**PVS volume (% in ROI)**	**PVS count**	**PVS density (count per ROI volume (in mm³))**	**PVS median length (mm)**
Fixed Effect								
**Intercept**	-0.04 (0.04)	-0.02 (0.04)	-0.02 (0.04)	-0.04 (0.04)	-0.08 (0.04)	-0.05 (0.04)	-0.05 (0.04)	-0.08 (0.04)
**Age (years)**	**0.18 (0.04), p< 0.001, [0.11 0.26]**	**0.12 (0.04), p=0.005, [0.04 0.2]**	**0.16 (0.04), p<0.001, [0.07 0.24]**	**0.15 (0.04), p<0.001, [0.07 0.23]**	**0.16 (0.04), p<0.001, [0.09 0.24]**	0.03 (0.04), p=0.48, [-0.05 0.11]	**0.09 (0.04), p=0.03, [0.01 0.17]**	**0.09 (0.04), p=0.02, [0.02 0.17]**
**Hypertension (yes/no)**	**0.17 (0.08), p = 0.04, [0.01 0.33]**	0.15 (0.09), p=0.09, [-0.02 0.33]	**0.18 (0.09), p=0.04, [0.01 0.36]**	0.07 (0.08), p=0.38, [-0.09 0.24]	**0.35 (0.09), p<0.001, [0.18 0.51]**	**0.25 (0.09), p=0.007, [0.07 0.42]**	**0.26 (0.09), p=0.003, [0.09 0.44]**	**0.23 (0.08), p=0.01, [0.06 0.4]**
**Hair cortisol (log)**	**0.07 (0.03), p = 0.04, [0.00 0.14]**	**0.09 (0.04), p=0.02, [0.01 0.16]**	**0.09 (0 .04), p=0.02, [0.01 0.16]**	0.02 (0.04), p=0.57, [-0.05 0.09]	0.01 (0.04), p=0.88, [-0.07 0.08]	0.02(0.04), p=0.67, [-0.06, 0.09]	0.02 (0.04), p=0.55, [-0.05 0.1]	-0.06 (0.04), p=0.09, [-0.13 0.01]
**Grip strength (kg)**	**-0.09 (0.04), p = 0.01, [-0.16 -0.02]**	0.01 (0.04), p=0.8, [-0.07 0.09]	**-0.08 (0.04), p=0.04, [-0.16 -0.004]**	-0.04 (0.04), p=0.27, [-0.12 0.02]	-0.06 (0.04), p=0.13, [-0.13 0.02]	0.02 (0.04), p=0.68, [-0.06 0.1]	-0.06 (0.04), p=0.17, [-0.12 0.30]	-0.02 (0.04), p=0.56, [-0.1 0.05]
**QIDS score**		**0.09 (0.04), p=0.02,**	**0.1 (0.04), p=0.005,**	-0.05 (0.03), p=0.18,	**0.09 (0.03), p=0.01,**	**0.11 (0.04), p=0.004,**	**0.12 (0.04), p=0.001,**	-0.06 (0.03), p=0.11,

	**0.08 (0.03), p = 0.02, [0.01 0.15]**	**[0.01 0.16]**	**[0.03 0.16]**	[-0.11 0.02]	**[0.02 0.16]**	**[0.03 0.18]**	**[0.05 0.19]**	[-0.12 0.01]
**WMH burden within ICV (log)**	**0.13 (0.04), p = 0.002, [0.05 0.21]**	**0.13 (0.04), p=0.003, [0.05 0.22]**	**0.12 (0.04), p=0.008, [0.03 0.2]**	**0.1 (0.04), p=0.02, [0.02 0.18]**	0.08 (0.04), p=0.06, [0 0.16]	0.03 (0.05), p=0.56, [-0.06 0.11]	0.02 (0.04), p=0.59, [-0.06 0.11]	**0.14 (0.04), p<0.001, [0.05 0.22]**
Random Effect								
**Family ID**	**0.23 (0.10), p=0.01, [0.01 0.52]**	0.09 (0.12), p=0.23, [0.01 1.26]	0.11 (0.12), p=0.17, [0.01 0.89]	**0.29 (0.08), p=0.0004, [0.16 0.5]**	0.04 (0.11), p=0.37, [0.001 12.0]	m.o.	m.o.	0.07 (0.09), p=0.19, [0.01 0.75]

Legend: PVS: MRI-visible enlarged perivascular spaces, ROI: Region Of Interest, m.o.: model overfitting

Table S3. False Discovery Rate-corrected q values per brain region.

	Centrum Semiovale				***Basal Ganglia***			
	**PVS volume (% in ROI)**	**PVS count**	**PVS density**	**PVS median length (mm)**	**PVS volume (% in ROI)**	**PVS count**	**PVS density**	**PVS median length (mm)**
Fixed Effect								
**Age (years)**	**<0.001**	**0.02**	**<0.001**	**<0.001**	**<0.001**	0.64	*0.065*	**0.04**
**Hypertension (y/n)**	0.1	0.19	0.1	0.51	**<0.001**	**0.04**	**0.032**	**0.01**
**Hair cortisol (log)**	0.11	*0.068*	*0.068*	0.67	1	0.89	0.84	0.21
**Grip strength (kg)**	**0.03**	0.77	*0.068*	0.26	0.37	0.65	0.38	0.89
**QIDS score**	*0.054*	*0.054*	**0.028**	0.25	**0.04**	**0.02**	**0.01**	0.27
**Percentage of WMH volume in ICV (log)**	**0.01**	**0.014**	**0.028**	**0.033**	*0.065*	0.64	0.64	**<0.001**
Random effect								
**Family ID**	**0.033**	0.27	0.22	**0.002**	0.47	1	1	0.27

Legend: PVS: MRI-visible enlarged perivascular spaces, ROI: Region Of Interest

Table S4. Non-standardized results from the linear mixed effects models (β, standard error (SE), p-value, 95% CI)

	Centrum Semiovale				***Basal Ganglia***			
	**PVS volume (% in ROI)**	**PVS count**	**PVS density**	**PVS median length (mm)**	**PVS volume (% in ROI)**	**PVS count**	**PVS density**	**PVS median length (mm)**
Fixed Effect								
**Intercept**	0.55 (0.37)	449.52 (210.33)	3.08E-3 (1.44E-3)	4.8 (0.34)	1.5 (0.38)	206.81 (47.84)	3.35E-3 (0.09E-3)	5.48 (0.31)
**Age (years)**	**0.02 (0.005), p<0.001, [0.14 0.032]**	**7.65 (2.71), p=0.005, [2.34 12.95]**	**0.07E-3 (0.02E-3), p<0.001, [0.03E-3 0.1E-3]**	**0.02 (0.004), p<0.001, [0.008 0.025]**	**0.02 (0.005), p<0.001, [0.01 0.03]**	0.44 (0.62), p=0.478, [-0.77 1.65]	**0.24E-4 (0.11E-4), p=0.027, [2.75E-6 0.46E-4]**	**0.01 (0.004), p=0.011, [0.0023 0.018]**
**Hypertension (y/n)**	*0.18 (0.1), p=0.067, [-0.013 0.38]*	83.4 (56.8), p=0.14, [-27.88 194.73]	*0.7E-3 (0.39E-3), p=0.068,* [-0.05E-3 1.47E-3]	0.066 (0.09), p=0.47, [-0.11 0.24]	**0.40 (0.10), p<0.001, [0.20 0.60]**	**31.71 (12.91), p=0.014, [6.39 57.03]**	**0.62E-3 (0.23E-3), p=0.008, [0.16E-3 1.07E-3]**	**0.25 (0.08), p=0.002, [0.09 0.42]**
**Hair cortisol (log)**	*0.1 (0.06), p=0.079, [-0.011 0.21]*	**65.2 (31.83), p=0.041, [2.81 127.58]**	**0.44E-3(0 .22E-3), p=0.041, [0.02E-3 0.87E-3]**	0.023 (0.05), p=0.65, [-0.076 0.12]	-0.002 (0.057), p=0.97, [-0.11 0.11]	1.97 (7.27), p=0.79, [-12.28 16.22]	0.5E-4 (1.3E-4), p=0.69, [-0.2E-3 0.3E-3]	-0.08 (0.047), p=0.098, [-0.17 0.014]
**Grip strength (kg)**	**-0.01 (0.004), p=0.013, [-0.02 -0.002]**	0.72 (2.49), p=0.77, [-4.16 5.60]	**-0.04E-3 (0.02E-3), p=0.038, [-0.07E-3 -1.97E-6]**	-0.005 (0.004), p=0.226, [-0.012 0.003]	-0.0056 (0.0045), p=0.21, [-0.014 0.003]	0.37 (0.57), p=0.51, [-0.74 1.49]	-0.12E-4 (0.1E-4), p=0.23, [-0.3E-4 7.8E-6]	-0.001 (0.004), p=0.764, [-0.008 0.006]
**QIDS score**	**0.025 (0.01), p=0.026, [0.003 0.048]**	**14.11 (6.40), p=0.027, [1,57 26.65]**	**0.1E-3 (0.04E-3), p=0.009, [0.03E-3 0.2E-3]**	-0.013 (0.01), p=0.207, [-0.033 0.0072]	**0.028 (0.011), p=0.014, [0.0058 0.051]**	**4.15(1.45), p=0.004, [1.30 7.0]**	**0.83E-4 (0.26E-4), p=0.002, [0.32E-4 1.34E-4]**	-0.014 (0.009), p=0.147, [-0.032 0.005]

**Percentage of WMH volume in ICV (log)**	**0.13 (0.04), p=0.002, [0.047 0.20]**	**66.56 (22.46), p=0.003, [22.53 110.58]**	**0.4E-3 (0.15E-3), p=0.009, [0.098E-3 0.7E-3]**	**0.09 (0.04), p=0.014, [0.018 0.16]**	**0.089 (0.040), p=0.028, [0.0098 0.17]**	3.97 (5.1), p=0.436, [-6.023 13.97]	0.68E-4 (0.92E-4), p=0.46, [-1.12E-4 2.47E-4]	**0.12 (0.033), p<0.001, [0.05 0.18]**
Random effect								
**Family ID**	**0.35 (0.15), p=0.013, [0.15 0.81]**	34.9E3 (49.8E3), p=0.24, [2.13E3 57.2E4]	2.28E-6 (2.44E-6), p=0.17, [2.81E-7 1.85E-5]	**0.34 (0.096), p=0.0003, [0.198 0.596]**	0.084 (0.16), p=0.304, [0.0019 3.79]	m.o.	5.4E-14 (9.5E-14), p=1.0, [1.7E-15 1.7E-12]	0.094 (0.093), p=0.147, [0.013 0.65]

Legend: PVS: MRI-visible enlarged perivascular spaces, ROI: Region Of Interest, m.o.: model overfitting

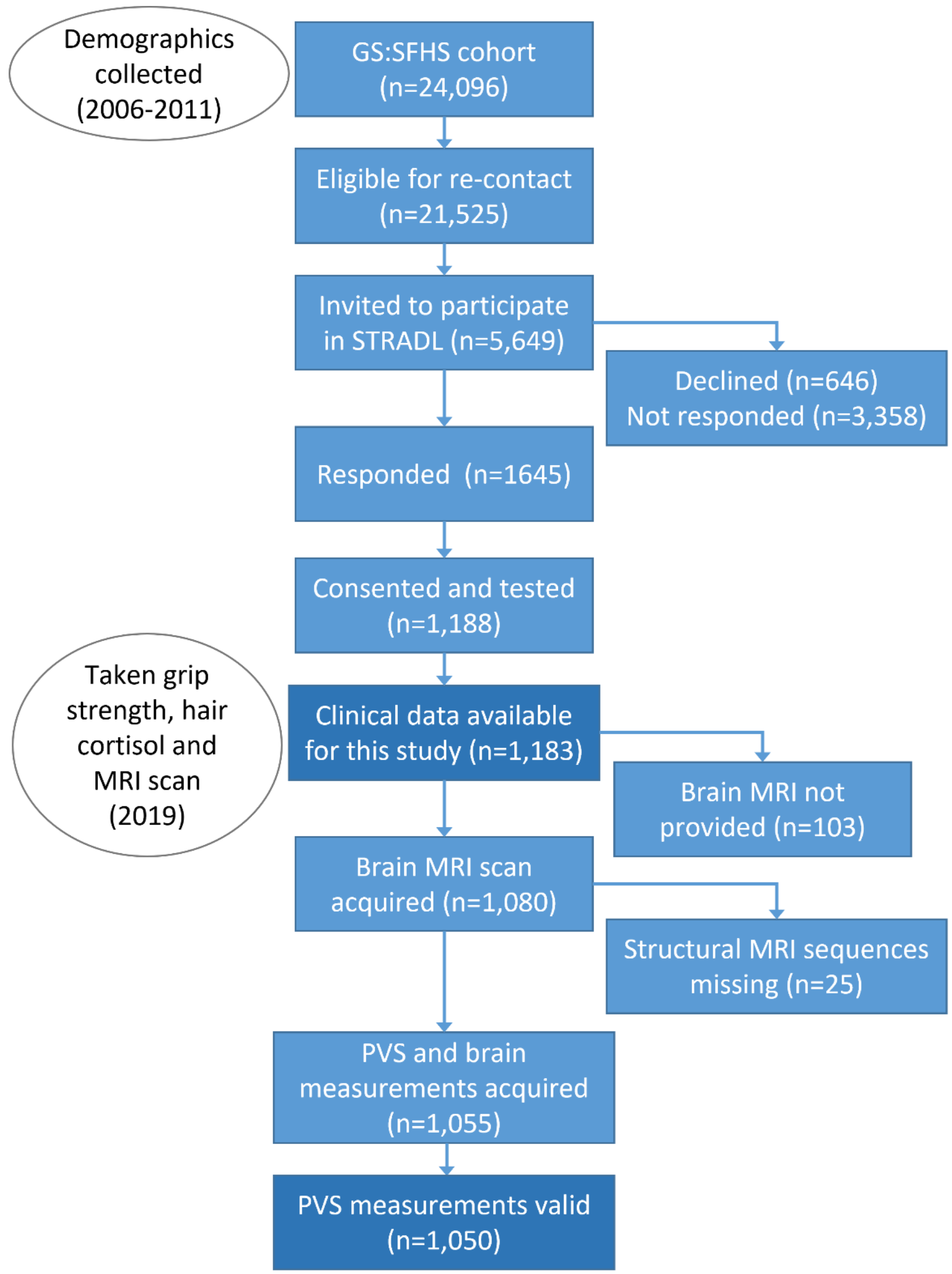

Figure S1. Flow chart of the recruitment and sample selection process.
